# Efficacy and safety of camrelizumab in combination with neoadjuvant chemotherapy for ESCC and its impact on esophagectomy

**DOI:** 10.3389/fimmu.2022.953229

**Published:** 2022-07-14

**Authors:** Yujin Qiao, Cong Zhao, Xiangnan Li, Jia Zhao, Qi Huang, Zheng Ding, Yan Zhang, Jia Jiao, Guoqing Zhang, Song Zhao

**Affiliations:** ^1^ Department of Thoracic Surgery, First Affiliated Hospital of Zhengzhou University, Zhengzhou, China; ^2^ Department of Nephrology, First Affiliated Hospital of Zhengzhou University, Zhengzhou, China

**Keywords:** esophageal squamous cell carcinoma (ESCC), camrelizumab, immune checkpoint inhibitor (ICI), neoadjuvant chemotherapy, efficacy, surgery

## Abstract

**Background:**

Esophageal squamous cell carcinoma (ESCC) is the most common type of esophageal cancer in China. The use of neoadjuvant immunotherapy for the treatment of ESCC is gradually increasing. Camrelizumab is one such immune checkpoint inhibitor (ICI) used for treatment. In this retrospective study, we explored the efficacy, safety, and short-term perioperative prognosis of camrelizumab in combination with neoadjuvant chemotherapy for ESCC.

**Materials and Methods:**

A total of 254 Chinese patients with ESCC were enrolled in the study; 48 received camrelizumab in combination with neoadjuvant chemotherapy (C-NC group), and 206 received neoadjuvant chemotherapy (NC group). All patients underwent surgery after the completion of 2 cycles of neoadjuvant therapy.

**Results:**

Twenty patients (20/48, 41.7%) in the C-NC group and 22 patients (22/206, 10.7%) in the NC group achieved a pathologic complete response (pCR) (p<0.001). Twenty-nine patients (29/48, 60.4%) in the C-NC group and 56 patients (56/206, 27.2%) in the NC group achieved major pathologic remission (MPR) (p<0.001). There was a lower incidence of myelosuppression during neoadjuvant therapy in patients in the C-NC group (33/48, 68.8%) than in the NC group (174/206, 84.5%, p=0.012). The total incidence of adverse reactions during neoadjuvant therapy was also lower in the C-NC group (37/48, 77.1%) than in the NC group (189/206, 91.7%, p=0.003). Patients in the C-NC group had more lymph nodes cleared during surgery than those in the NC group (34 *vs*.30, p<0.001). The logistic model showed that the treatment regimen, age, and presence of lymph node metastasis were influential factors for achieving a pCR in these patients (p<0.001). Regarding other adverse events and surgery-related data, there were no significant differences observed between the two groups.

**Conclusion:**

Camrelizumab in combination with neoadjuvant chemotherapy is an efficacious neoadjuvant regimen with an acceptable safety profile and does not increase the difficulty of surgery or the incidence of complications. A pCR is more likely to be achieved in patients treated with camrelizumab in combination with neoadjuvant chemotherapy, in younger patients, or in those without lymph node metastases.

## 1 Introduction

More than 540,000 people die from esophageal cancer each year, accounting for 5.5% of all cancer-related deaths (1). Esophageal cancer has a significant geographic distribution worldwide. Asia accounts for the majority of esophageal cancer cases worldwide, with 49% of the cases occurring in China (1, 2). Esophageal squamous cell carcinoma (ESCC) is the most common pathological type in cases of Asian origin, as well as worldwide (3, 4).

The treatment of esophageal cancer normally includes single or combined treatments, including surgery, chemotherapy, radiotherapy, targeted therapy and immunotherapy (4–6). Esophageal cancer is difficult to detect until progression or distant metastasis occurs, and more than 40% of patients already have distant metastasis at the time of diagnosis (2, 4). Due to the complex anatomical structure around the esophagus, surgical treatment alone increases the risk of incomplete tumor removal, potentially increasing the risk of local recurrence, and its therapeutic effect is not satisfactory (4, 6). Neoadjuvant therapy can shrink the tumor size, reduce the pathological stage, and eliminate the potential presence of subclinical micrometastases (7–9). Several studies have shown that neoadjuvant chemotherapy improves the prognosis of patients compared to surgery alone (10–13).

In recent years, with the advent and use of immunotherapy (5), it has been shown that immunotherapy does not significantly increase adverse effects compared to chemotherapy (14–17). Therefore, doctors have begun to explore whether immune checkpoint inhibitors (ICIs) combined with neoadjuvant chemotherapy can achieve better efficacy and safety than neoadjuvant chemotherapy. At the same time, because esophagectomy and lymph node dissection are very complex and involve multiple operative areas, various complications can arise during the perioperative period and are major concerns for clinicians. It has been demonstrated that preoperative neoadjuvant therapy does not affect postoperative quality of life compared to surgery alone (18–20); therefore, we also wanted to explore whether surgery following the use of camrelizumab increases the difficulty of surgery or the incidence of complications.

Camrelizumab is an ICI developed by a Chinese pharmaceutical company that targets PD-1. This study explored the clinical efficacy and safety of camrelizumab combined with neoadjuvant chemotherapy for the treatment of ESCC and recorded data related to surgery and the occurrence of various complications during the perioperative period, which are reported below.

## 2 Materials and methods

### 2.1 Patients

**Figure 1:**
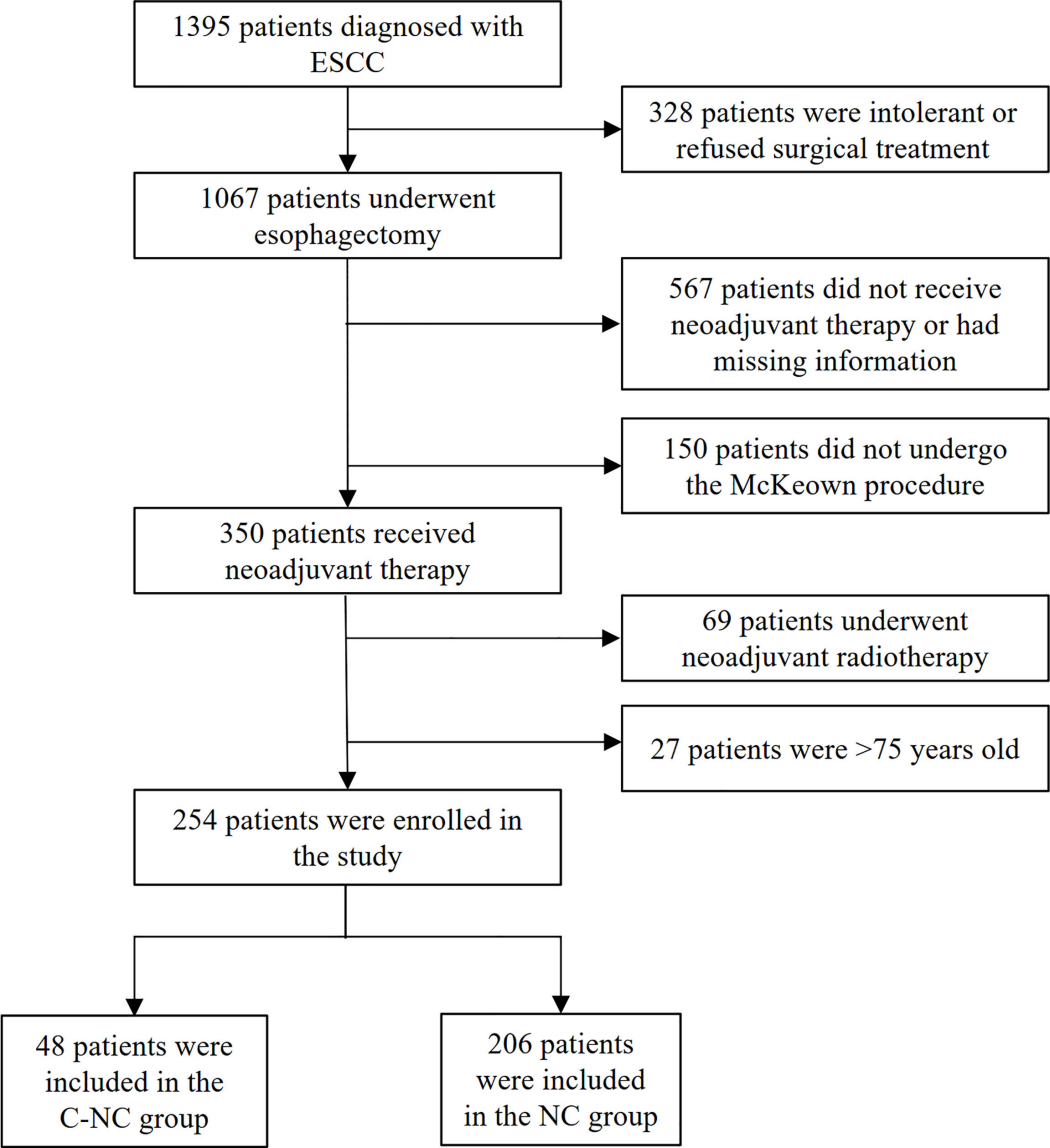
The participant selection algorithm.

In this retrospective single-center study, we collected data from esophageal cancer patients who underwent surgery at the First Affiliated Hospital of Zhengzhou University from January 2019 to December 2021. The inclusion criteria were as follows: I. diagnosis of esophageal cancer based on preoperative pathological examination, with a pathological type of squamous cell carcinoma; II. diagnosis based on standardized physical examination and imaging examination, with a clinical stage of cT_1-4a_N_0-3_M_0_ determined by computed tomography (CT) and endoscopic ultrasonography (EUS); III. administration of 2 cycles of neoadjuvant chemotherapy combined with camrelizumab or neoadjuvant chemotherapy alone; IV. treatment with a minimally invasive McKeown procedure (trans-right thoracic + trans-abdominal + cervical anastomosis), achieving R0 resection; V. age ≤75 years, with a good general condition and normal cardiopulmonary and other organ function; and VI. well-documented medical records. The exclusion criteria were as follows: I. concomitant malignant tumors of another type; and II. contraindications to surgery or inability to tolerate surgery. A total of 254 patients were enrolled in the study and were divided into two groups according to the neoadjuvant regimen used: the C-NC group (patients received 200 mg of camrelizumab combined with neoadjuvant chemotherapy, n=48) and the NC group (patients received neoadjuvant chemotherapy, n=206) (**Figure 1**). This research was approved by the Ethics Committee of the First Affiliated Hospital of Zhengzhou University.

### 2.2 Treatment

All patients received a professional and adequate medical examination and evaluation before receiving treatment, and EUS and pathological biopsy of the primary tumor were completed before treatment. Hydration, antiemetics, and hepatoprotection were routinely administered during neoadjuvant therapy.

#### 2.2.1 Camrelizumab

Patients were administered 200 mg of camrelizumab on the first day of neoadjuvant treatment every 3 weeks. Patients were excluded if they had an active autoimmune or infectious disease, if they were undergoing systemic corticosteroid or other immunosuppressive therapy, if they were allergic to camrelizumab, or if immunotherapy could not be administered due to a serious adverse event.

#### 2.2.2 Neoadjuvant chemotherapy

All patients received full doses of the neoadjuvant chemotherapy regimen (platinum-containing double-drug chemotherapy regimen including paclitaxel, albumin-bound paclitaxel or docetaxel) recommended by the Chinese Society of Clinical Oncology (CSCO) guidelines or according to the specific research protocols. Patients were removed from the study if the dose was reduced or discontinued because the neoadjuvant chemotherapy could not be tolerated.

#### 2.2.3 Operation

The surgical approach used for all patients was a minimally invasive McKeown’s procedure (trans-right thoracic + trans-abdominal + cervical anastomosis), in which lymph nodes around the esophagus and stomach were routinely cleared. The decisions to clear the cervical lymph nodes were made based on the results of pathological frozen sections of the laryngeal nerve lymph nodes (21). All procedures were performed by the same team of surgeons. Negative cut margins were observed. R0 resection was defined as the absence of cancer cells on the cut margins as observed by the naked eye and pathological section examination.

### 2.3 Pathology and adverse events

Pathological examination of the surgically removed tissue specimens was performed, and the tumor and lymph nodes were independently evaluated by two pathologists from our hospital after neoadjuvant treatment. The histological type of esophageal cancer was determined according to the 2019 edition of the World Health Organization (WHO) Classification of Tumors of the Digestive System. The tumor regression grade (TRG) was based on the criteria of the College of American Pathologists (CAP), which is in line with the approach recommended by the CSCO/National Comprehensive Cancer Network (NCCN) guidelines (19) for the management of esophageal cancer. The TNM stage was determined according to the International Union Against Cancer (UICC)/American Joint Committee on Cancer (AJCC) TNM Staging System (8th edition, 2017) (22). Adverse events occurring during neoadjuvant therapy and during the perioperative period were recorded and graded according to the Common Terminology Criteria for Adverse Events (CTCAE) version 5.0 published by the National Cancer Institute (23). Myelosuppression was defined as a decrease in the activity of blood cell precursors in the bone marrow, and the diagnostic criteria were white blood cell, granulocyte, hemoglobin, and platelet counts less than the lower limit of normal.

### 2.4 Outcomes

After undergoing neoadjuvant therapy and surgery, the primary clinical endpoint was the pathological response of the primary tumor, and secondary clinical endpoints included surgery-related data, the postoperative hospital stay, and adverse events during neoadjuvant therapy and during the perioperative period. Patients with primary tumor regression rated as TRG=0 and without lymph node metastasis (ypT0N0) were considered to have achieved a pathologic complete response (pCR), those with TRG=0 or 1 were considered to have achieved major pathologic remission (MPR), and those with TRG=2 or 3 were considered to have achieved nonsignificant or nonresponsive remission. The relationship between the treatment effect and TRG is shown in **Table 1**. The lymph node response was evaluated based on postoperative pathology, including whether metastasis or regression had occurred.

**Table 1:** Relationship between the treatment effect and tumor regression grade (TRG).

Treatment effect	TRG
Present, with no viable cancer cells	Complete response, score of 0
Present, with single cells or rare small groups of cancer cells	Near complete response, score of 1
Present, with residual cancer showing evident tumor regression, but more than single cells or rare small groups of cancer cells	Partial response, score of 2
Absent, with extensive residual cancer and no evident tumor regression	Poor or no response, score of 3

### 2.5 Statistical analysis

Statistical analysis was performed using the statistical software SPSS 24.0 (SPSS, Inc., Chicago, IL, USA). Parametric data are expressed as the means ± standard deviations (SDs). Nonparametric data are expressed as the median [interquartile range (IQR)]. The extent of tumor regressionis expressed as a frequency or percentage and 95% confidence interval (CI). A t test was used for the analysis of normally distributed data in different groups. The Kruskal–Wallis test or the Mann–WhitneyU test was used for the analysis of semiquantitative data. The chi-squared test or Fisher’s exact test was used for the analysis of qualitative data. All probabilities were two-tailed, and the level of significance was set at 0.05.

## 3 Results

### 3.1 Basic information

The mean age of the participants in the two groups (C-NC vs. NC) was 64.15 ± 7.293 vs. 62.22 ± 7.136 years. There were 38 vs. 147 males and 10 vs. 59 females in these two groups(C-NC vs. NC). The median body mass index (BMI) was 23.70 (IQR 22.05, 25.35) vs. 23.21(IQR 21.20, 25.22), respectively (C-NC vs. NC). There were 45 patients with upper thoracic esophageal cancer (8 vs. 37, C-NC vs. NC), 127 patients with mid-thoracic esophageal cancer (26 vs. 101, C-NC vs. NC), and 82 patients with lower thoracic esophageal cancer (14 vs. 68, C-NC vs. NC). Any history of smoking, alcohol consumption, and any past history of smoking/drinking were also recorded. All baseline characteristics in the two groups were comparable, with no significant differences observed (**Table 2**).

**Table 2:** Clinical characteristics of the patients with ESCC treated with different neoadjuvant therapies.

	Groups		
Characteristics	C-NC (n=48)	NC (n=206)	*P*
**Age, y, Mean (SD)**	64.15 ± 7.293	62.22 ± 7.136	0.095
**Sex, n (%)**			0.273
Male	38 (79.2)	147 (71.4)	
Female	10 (20.8)	59 (28.6)	
**BMI, M(IQR)**	23.70 (22.05,25.35)	23.21 (21.20,25.22)	0.466
**Tumor location, n (%)**			0.810
Upper	8 (16.7)	37 (18.0)	
Middle	26 (54.2)	101 (49.0)	
Lower	14 (29.2)	68 (33.0)	
**Smoking, n (%)**			0.185
Yes	27 (56.3)	94 (45.6)	
No	21 (43.8)	112 (54.4)	
**Drinking, n (%)**			0.107
Yes	20 (41.7)	61 (29.6)	
No	28 (58.3)	145 (70.4)	
**Past history, n (%)**			0.215
Yes	19 (39.6)	102 (49.5)	
No	29 (60.4)	104 (50.5)	

C-NC, camrelizumab+ neoadjuvant chemotherapy; NC, neoadjuvant chemotherapy; BMI, body mass index. The tumor location is marked according to the location of the midpoint of the tumor.

### 3.2 Postoperative pathology

A pCR of the primary tumor was achieved in 20 of 48(41.7%) patients in the C-NC group vs. 22 of 206(10.7%) patients in the NC group (p<0.001). To better demonstrate the effect of neoadjuvant therapy, we also compared the MPR rates in the C-NC and NC groups, and the C-NC group showed encouraging results: 29 of 48(60.4%) vs. 56 of 206(27.2%) patients achieved MPR (C-NC vs. NC, p<0.001). Pathologists also considered the lymph node response, such as granuloma formation, necrosis, and fibrosis, to neoadjuvant therapy, and no significant differences were found between the two groups (p=0.102) (**Table 3**). Additional pathological information is presented in **Table 3**.

**Table 3:** Postoperative pathology of patients with ESCC.

	Groups				
	C-NC(n=48)	NC(n=206)	χ^2^	P	OR (95% CI)
**pCR**			27.084	<0.001	5.974 (2.895,12.327)
Yes	20 (41.7)	22 (10.7)			
No	28 (58.3)	184 (89.3)			
**MPR**			19.309	<0.001	4.088 (2.124,7.870)
Yes	29 (60.4)	56 (27.2)			
No	19 (39.6)	150 (72.8)			
**LN response**			2.669	0.102	0.357 (0.123,1.037)
Yes	6 (12.5)	10 (4.9)			
No	42 (87.5)	196 (95.1)			
**TRG**					
0	21 (43.8)	34 (16.5)			
1	8 (16.7)	22 (10.7)			
2	8 (16.7)	36 (17.5)			
3	11 (22.9)	114 (55.3)			
**T stage**					
0	21 (43.7)	35 (17.0)			
1	10 (20.8)	28 (13.6)			
2	9 (18.8)	65 (31.6)			
3	8 (16.7)	78 (37.9)			
**N stage**					
0	30(62.5)	104(50.5)			
1	9(18.8)	59(28.6)			
2	7(14.6)	37(18.0)			
3	2(4.2)	6(2.9)			

TRG, tumor regression grade; pCR, pathologic complete response; MPR, major pathological remission; LN, lymph node.

### 3.3 Adverse events and perioperative data

In total, 33 of 48(68.8%) patients in the C-NC group suffered from varying degrees of myelosuppression during neoadjuvant therapy compared to 174 of 206(84.5%) in the NC group. Significant differences were found in terms of the incidence of myelosuppression between the two groups (p=0.012). Regarding liver and kidney function injury, gastrointestinal reactions, cardiovascular events and skin damage during neoadjuvant therapy, the incidences did not differ statistically between the two groups (**Table 4**). Most adverse reactions during neoadjuvant therapy were mild and manageable and did not interfere with neoadjuvant therapy or subsequent surgery after observation or drug treatment. Grade 3 or higher myelosuppression occurred in 3 patients in the C-NC group and in 16 patients in the NC group. Only 1 patient in the C-NC group developed grade 3 liver function injury. One patient in the NC group developed acute kidney injury (grade 4), and after hydration and 2 hemodialysis sessions, this patient’s kidney function returned to normal. Although there was a high incidence of reactive cutaneous capillary endothelial proliferation (RCCEP), which is usually associated with camrelizumab, all recorded cases were grade 1 or 2 (26 of 48, 54.2%).

**Table 4:** Adverse events during neoadjuvant therapy and the perioperative period and perioperative data.

	Groups				
	C-NC(n=48)		NC(n=206)	χ^2^	P
**Myelosuppression (all grades), n (%)**	33(68.8)		174(84.5)	6.376	0.012
**Liver function injury (all grades), n (%)**	17(35.4)		78(37.9)	0.100	0.752
**Kidney function injury (all grades), n (%)**	0(0.0)		4(1.9)		>0.999
**Gastrointestinal reaction, n (%)**	6(12.5)		19(9.2)	0.174	0.676
**Cardiovascular events, n (%)**	0(0.0)		2(1.0)		>0.999
**Skin damage, n (%)**	2(4.2)		1(0.5)		0.093
**RCCEP, n (%)**	26(54.2)		–		–
**Total^a^, n (%)**	37(77.1)		189(91.7)	8.535	0.003
**Blood transfusion, n (%)**	1(2.1)		21(10.2)	2.293	0.130
**Surgical injury, n (%)**	0(0.0)		7(3.4)	0.649	0.420
**PPCs, n (%)**	13(27.1)		77(37.4)	1.804	0.179
**EGAF, n (%)**	4(8.3)		10(4.9)	0.360	0.549
**ICU, n (%)**	4(8.3)		7(3.4)	1.252	0.263
**Arrhythmia, n (%)**	0(0.0)		14(6.8)	2.271	0.132
**Delayed incision healing, n (%)**	0(0.0)		3(1.5)		>0.999
**Total^b^, n (%)**	17(35.4)		99(48.1)	2.507	0.113
**Operation time, min, M(IQR)**	324.5(264.0,385.0)		310.0(265.0,355.0)		0.595
**Postoperative hospital stay, d, M(IQR)**	10(8.5,11.5)		9(6.5,11.5)		0.704
**Number of dissected lymph nodes, M(IQR)**	34(28.5,39.5)		30(26,34)		<0.001

All grades of adverse events were recorded in this table, including death. Postoperative hospital stay was counted from the first day after surgery and ended on the day of discharge. RCCEP: reactive cutaneous capillary endothelial proliferation; PPCs: postoperative pulmonary complication, including pneumonia, atelectasis and pleural effusion; EGAF: esophagogastric anastomotic fistula. Total^a^ indicates the total adverse event rate during neoadjuvant therapy. Total^b^ indicates the total adverse event rate in the perioperative period.

The operation time was 324.5(IQR 264.0, 385.0) minutes vs. 310.0(IQR 265.0, 355.0) minutes(C-NC vs. NC, p=0.595). The number of dissected lymph nodes was 34(IQR 28.5, 39.5) in the C-NC group and 30(IQR 26.0, 34.0) in the NC group, with a significant difference (p<0.001). No significant differences were found between the two groups in terms of blood transfusions, surgical injuries, postoperative pulmonary complications (PPCs), esophagogastric anastomotic fistula formation, unexpected transfer to the ICU, cardiac arrhythmias, or delayed incision healing (p>0.05); the relevant data are shown in **Table 4**. Of the seven patients in the NC group who were unexpectedly transferred to the ICU, three eventually died on days 1, 1, and 7 after transfer to the ICU, all due to cardiac arrest, and one of these patients had a combined esophagogastric anastomotic fistula. The other patients were successfully transferred back to the general ward.

### 3.4 Benefit population

We analyzed the clinical data of patients who did and did not achieve a pCR to determine which factors were more likely to benefit patients. In this study, binary logistic regression was used to assess whether treatment, sex, age, tumor location, BMI, smoking history, alcohol consumption, past history, and lymph node metastasis affected the pCR rate. Four observations with studentized residuals greater than 2.5 times the SD were retained in the analysis. Ultimately, the obtained logistic model was significant (χ2 = 36.359, p<0.001). The model was able to correctly classify 78.3% of the study subjects. Of the nine variables included in the model, the use of camrelizumab in combination with neoadjuvant chemotherapy, a younger age, and no lymph node metastases increased the likelihood of patients achieving a pCR (**Table 5**).

**Table 5:** Logistic model of factors increasing the likelihood of patients achieving a pCR.

	Wald	P	OR(95% CI)
Camrelizumab + NC	16.347	<0.001	4.805(2.245, 10.283)
Sex	0.532	0.466	1.368(0.590, 3.172)
Younger age	4.991	0.025	1.054(1.007, 1.104)
BMI	1.439	0.230	0.936(0.841, 1.043)
Tumor location			
Upper	Baseline	0.990	Baseline
Middle	0.014	0.906	0.944(0.364, 2.449)
Lower	0.000	0.988	1.006(0.468, 2.161)
Smoking	0.329	0.567	1.283(0.548, 3.005)
Drinking	0.223	0.637	1.236(0.513, 2.974)
Past history	1.967	0.161	0.613(0.309, 1.215)
NoLN metastases	10.638	0.001	3.295(1.609, 6.742)

NC, neoadjuvant chemotherapy; LN, lymph nodes. P<0.05 is considered to indicate a significant difference.

## 4 Discussion

In this retrospective study, we explored the safety and efficacy of camrelizumab in combination with neoadjuvant chemotherapy and explored what populations might benefit from treatment. Some information on these patients in the perioperative period was also recorded to analyze the possible impact of neoadjuvant therapy on surgery.

Camrelizumab is a human IgG4 monoclonal antibody that targets PD-1 with a strong affinity and inhibits the binding of PD-L1/PD-L2 to PD-1. It has been used to treat unresectable advanced ESCC with acceptable efficacy and safety profiles (5, 6, 15, 16, 24, 25). However, its safety and efficacy are still in the exploratory stage in terms of its application in a neoadjuvant therapy setting (26–31).

A phase II clinical study by Liu J et al. (27) explored the efficacy and adverse events of camrelizumab in combination with neoadjuvant chemotherapy. The final results showed that 39.2% (20 of 51) of the patients ultimately achieved a pCR, which is similar to the results we observed. This is an exciting result that demonstrates the potential of camrelizumab in a neoadjuvant therapy setting. Meanwhile, the choice of neoadjuvant chemotherapy regimen in this study differed and was not identical to that in the studies by Liu J and Yang Y et al. (27, 30). However, we obtained similar pCR rates, indicating that the choice of chemotherapy regimen may not affect the final resolution for patients on camrelizumab. More work is needed to confirm this speculation.

We also reviewed several studies on the use of ICIs in neoadjuvant therapy. Liu J et al. (26) used a neoadjuvant regimen consisting of camrelizumab, nab-paclitaxel and cisplatin, and the final pCR rate was 35.3%. Yang G et al. (28) used a neoadjuvant regimen consisting of camrelizumab plus nab-paclitaxel and S1 capsules, and the final pCR rate was 33.3% (4/12). Yang W et al. (29) used a neoadjuvant regimen consisting of camrelizumab, nab-paclitaxel, and carboplatin for esophageal cancer, and the final pCR rate was 25% (5/23). Yang P et al. (31) used a neoadjuvant regimen consisting of camrelizumab, paclitaxel and carboplatin, and the final pCR rate was 31.3%. Other studies of ICIs (PD-1) in combination with neoadjuvant chemotherapy are shown in **Table 6**. Overall, regimens with camrelizumab demonstrated better pCR rates.

**Table 6:** Studies of ICIs (PD-1) in combination with neoadjuvant chemotherapy.

Researchers	Institution	Year	Pathology	Stage	Treatment options	Number of participants	pCR rate
Wu Z et al. (32)	Fudan University Shanghai Cancer Center	2021	SCC	III-IVb	Pembrolizumab or camrelizumab	38	34.21%
Shang X et al. (33, 34)	Tianjin Medical University Cancer Institute and Hospital	2022	SCC	II-III	Pembrolizumab + paclitaxel + cisplatin	–	–
Huang B et al. (35)	Fifth Affiliated Hospital of Sun Yat-sen University	2021	SCC	II-IVa	Pembrolizumab + docetaxel + nedaplatin	23	30.4%
Gao L et al. (36)	Fujian Medical University Union Hospital	2022	SCC	II-IVa	Toripalimab + docetaxel + cisplatin	12	16.7%
Zheng Y et al. (37, 38)	Affiliated Cancer Hospital of Zhengzhou University	2021	SCC	Ia-III	Toripalimab + paclitaxel + cisplatin	–	–
He W et al. (39)	Sichuan Cancer Hospital and Research Institute	2022	SCC	III-IVa	Toripalimab + paclitaxel + carboplatin	16	18.8%
Zhang Z et al. (40)	Fujian Medical University Union Hospital	2021	SCC	II-III	Sintilimab + albumin-bound paclitaxel + cisplatin	30	21.7%

Since the degree of lymph node regression is not the same as that of primary tumor regression (41), it has been suggested that some of the lymph node changes may not be related to neoadjuvant therapy (42). Therefore, the regression of lymph nodes was not described or explored in depth in this study.

We found that younger patients or those without lymph node metastases were more likely to achieve a pCR in this study. The determination of lymph node metastasis was based on postoperative pathology, as the status of lymph node metastasis is difficult to evaluate before surgery. Additionally, younger patients are more likely to have a better prognosis (43–45). The absence of lymph node metastases also means that the disease is still in an early stage, which could suggest a better prognosis. In a study involving 1,792 patients with esophageal cancer, Zhang GQ et al. (46) found that among patients without lymph node metastases, clearing more lymph nodes in younger patients could result in a better prognosis than in older patients, which is consistent with the conclusion we reached.

In terms of safety during neoadjuvant therapy, camrelizumab also demonstrated surprising results. Both in terms of the phenomena observed during actual clinical treatment and the results of the final statistical analysis, all grades of adverse events of camrelizumab in combination with neoadjuvant chemotherapy appeared to be less than those of neoadjuvant chemotherapy alone (77.1% vs. 91.7%). This proportion was also smaller than that in other studies (26, 27). In patients in the C-NC group, the majority of adverse events during neoadjuvant therapy were not severe (grade 1 or 2), and no grade 4 or higher adverse events were observed. The most common adverse events were leukopenia, lymphopenia, decreased hemoglobin, and occasionally thrombocytopenia. In general, the incidence of myelosuppression in the C-NC group remained lower than that in the NC group. We did not find any studies in the literature to explain this phenomenon, which may be related to differences in chemotherapy regimens and appropriate reductions in the dose of chemotherapy drugs when administering camrelizumab or to the preference to administer this treatment to more physically robust patients. This may also be related to bias from a small sample size or case screening. This phenomenon still needs to be explored in further large-sample analyses.

However, although the incidence of gastrointestinal reactions and skin damage after immunotherapy in this study was not significantly different from that after neoadjuvant chemotherapy, we observed that the incidence was higher (12.5% vs. 9.2%, and 4.2% vs. 0.5%). We believe that this finding deserves more attention and needs to be explored in a larger-sample study.

In our experience, compared to surgery alone, performing surgery after neoadjuvant treatment is typically more difficult, and the incidence of postoperative complications isincreased. Hong ZN et al. (47) also confirmed this in their study. Naidoo J et al. (48) reported that the response of the tumor and surrounding tissue to neoadjuvant therapy can lead to dense fibrosis, which poses a technical challenge for dissecting the tissue. In this study, we observed that in patients treated with camrelizumab, we were able to dissect more lymph nodes (34 vs. 30, p<0.001) within a similar surgical duration, which is beneficial for more accurate lymph node staging and for improving patient prognosis (19, 46, 49, 50).

Surgery-related injuries in these patients are commonly associated with damage to the thoracic duct and the recurrent laryngeal nerve and are often detected only after the manifestation of symptoms, such as chylothorax, hoarseness and choking while drinking water. Pneumonia, atelectasis or pleural effusion on the nonoperative side (left) account for most PPCs. The occurrence of PPCs may be related to the length of chest surgery, and dense fibrosis may prolong the surgery (47, 48). The incidences of esophagogastric anastomotic fistula formation and transfer to the ICU in the present study were not significant; however, they were the most dangerous and serious postoperative complications. All four of these factors, as well as delayed incision healing, were associated with significantly longer postoperative hospital stays.

Although postoperative complications were observed, the overall incidence was low, and they were manageable, with no deaths occurring in the C-NC group. Based on the experience of and studies by Hong ZN et al. (47, 51, 52), esophagectomy after neoadjuvant therapy is safe and feasible. Therefore, we consider the challenges and risks of surgical treatment after neoadjuvant immunotherapy to be acceptable.

Limitations: This study has some limitations. Due to theretrospective study design, the risks of selection bias and information bias are inevitable, and although we collected patient data as comprehensively as possible, these potential biases could not be eliminated. Only single-center data were collected, but the sample size was limited. A large multicenter study is needed to validate the findings.

## 5 Conclusion

Camrelizumab in combination with neoadjuvant chemotherapy has been shown to be more effective than neoadjuvant chemotherapy, and its safety profile and impact on surgery are satisfactory. A pCR is more likely to be achieved in patients treated with camrelizumab in combination with neoadjuvant chemotherapy, in younger patients, or in those without lymph node metastases.

## Data availability statement

The original contributions presented in the study are included in the article/supplementary material. Further inquiries can be directed to the corresponding author/s.

## Ethics statement

This study was reviewed and approved by The First Affiliated Hospital of Zhengzhou University Ethics Review Committee. Written informed consent for participation was not required for this study in accordance with the national legislation and the institutional requirements.

## Author contributions

SZ conceived of the idea and provided guidance. YQ and CZ wrote the manuscript and completed the tables and figure. XL and JZ contributed to organizing the database. QH, YZ, ZD, and JJ carefully reviewed the manuscript. GZ made critical revisions to the manuscript. All authors contributed to the article and approved the submitted version.

## Conflict of interest

The authors declare that the research was conducted in the absence of any commercial or financial relationships that could be construed as a potential conflict of interest.

## Publisher’s note

All claims expressed in this article are solely those of the authors and do not necessarily represent those of their affiliated organizations, or those of the publisher, the editors and the reviewers. Any product that may be evaluated in this article, or claim that may be made by its manufacturer, is not guaranteed or endorsed by the publisher.

## References

[1] Sung H Ferlay J Siegel RL Laversanne M Soerjomataram I Jemal A. Global cancer statistics 2020: globocan estimates of incidence and mortality worldwide for 36 cancers in 185 countries. CA Cancer J Clin (2021) 71(3):209–49. doi: 10.3322/caac.21660 33538338

[2] Uhlenhopp DJ Then EO Sunkara T Gaduputi V. Epidemiology of esophageal cancer: update in global trends, etiology and risk factors. Clin J Gastroenterol (2020) 13(6):1010–21. doi: 10.1007/s12328-020-01237-x 32965635

[3] Umar SB Fleischer DE Esophageal cancer: epidemiology, pathogenesis and prevention. Nat Clin Pract Gastroenterol Hepatol (2008) 5(9):517–26. doi: 10.1038/ncpgasthep1223 18679388

[4] Noordman BJ VM Lagarde SM Hulshof MCCM van Hagen P van Berge Henegouwen MI Wijnhoven BPL. Management of patients with adenocarcinoma or squamous cancer of the esophagus. Gastroenterology (2018) 154(2):437–51. doi: 10.1053/j.gastro.2017.09.048 29037469

[5] Zhao Q Yu J Meng X. A good start of immunotherapy in esophageal cancer. Cancer Med (2019) 8(10):4519–26. doi: 10.1002/cam4.2336 PMC6712478 31231980

[6] Watanabe M Otake R Kozuki R Toihata T Takahashi K Okamura A. Recent progress in multidisciplinary treatment for patients with esophageal cancer. Surg Today (2020) 50(1):12–20. doi: 10.1007/s00595-019-01878-7 31535225 PMC6952324

[7] Cunningham D Allum W Stenning S Thompson J Van de Velde C Nicolson M. Perioperative chemotherapy versus surgery alone for resectable gastroesophageal cancer. New Engl J Med (2006) 355(1):11–20. doi: 10.1056/NEJMoa055531 16822992

[8] Topalian SL Taube JM Pardoll DM. Neoadjuvant checkpoint blockade for cancer immunotherapy. Science (2020) 367(6477):eaax0182. doi: 10.1126/science.aax0182 32001626 PMC7789854

[9] Urschel JD Vasan H. A Meta-Analysis of Randomized Controlled Trials That Compared Neoadjuvant Chemoradiation and Surgery to Surgery Alone for Resectable Esophageal Cancer. The American Journal of Surgery (2003) 185(6):538–43. doi: 10.1016/s0002-9610(03)00066-7 12781882

[10] Law S FM Chow S Chu KM Wong J. Preoperative chemotherapy versus surgical therapy alone for squamous cell carcinoma of the esophagus : a prospective randomized trial. J Thorac Cardiovasc Surg (1997) 114(2):210–7. doi: 10.1016/S0022-5223(97)70147-8 9270638

[11] Ychou M Boige V Pignon JP Conroy T Bouche O Lebreton G. Perioperative chemotherapy compared with surgery alone for resectable gastroesophageal adenocarcinoma: an fnclcc and ffcd multicenter phase iii trial. J Clin Oncol (2011) 29(13):1715–21. doi: 10.1200/JCO.2010.33.0597 21444866

[12] Ando N Kato H Igaki H Shinoda M Ozawa S Shimizu H. A randomized trial comparing postoperative adjuvant chemotherapy with cisplatin and 5-fluorouracil versus preoperative chemotherapy for localized advanced squamous cell carcinoma of the thoracic esophagus (jcog9907). Ann Surg Oncol (2012) 19(1):68–74. doi: 10.1245/s10434-011-2049-9 21879261

[13] Ronellenfitsch U Schwarzbach M Hofheinz R Kienle P Kieser M Slanger TE. Preoperative chemo(radio)therapy versus primary surgery for gastroesophageal adenocarcinoma: systematic review with meta-analysis combining individual patient and aggregate data. Eur J Cancer (2013) 49(15):3149–58. doi: 10.1016/j.ejca.2013.05.029 23800671

[14] Shen D Chen Q Wu J Li J Tao K Jiang Y. The safety and efficacy of neoadjuvant pd-1 inhibitor with chemotherapy for locally advanced esophageal squamous cell carcinoma. J Gastrointest Oncol (2021) 12(1):1–10. doi: 10.21037/jgo-20-599 33708420 PMC7944149

[15] Zhang B Qi L Wang X Xu J Liu Y Mu L. Phase ii clinical trial using camrelizumab combined with apatinib and chemotherapy as the first-line treatment of advanced esophageal squamous cell carcinoma. Cancer Commun (Lond) (2020) 40(12):711–20. doi: 10.1002/cac2.12119 PMC7743020 33314747

[16] Zhang W Yan C Gao X Li X Cao F Zhao G. Safety and feasibility of radiotherapy plus camrelizumab for locally advanced esophageal squamous cell carcinoma. Oncologist (2021) 26(7):e1110–e24. doi: 10.1002/onco.13797 PMC8265339 33893689

[17] Hirano H Kato K. Systemic treatment of advanced esophageal squamous cell carcinoma: chemotherapy, molecular-targeting therapy and immunotherapy. Jpn J Clin Oncol (2019) 49(5):412–20. doi: 10.1093/jjco/hyz034 30920626

[18] Noordman B Verdam M Lagarde S Hulshof M van Hagen P van Berge Henegouwen M. Effect of neoadjuvant chemoradiotherapy on health-related quality of life in esophageal or junctional cancer: results from the randomized cross trial. J Clin Oncol Off J Am Soc Clin Oncol (2018) 36(3):268–75. doi: 10.1200/jco.2017.73.7718 29161204

[19] Ajani JA D'Amico TA Bentrem DJ Chao J Corvera C Das P. Esophageal and esophagogastric junction cancers, version 2.2019, nccn clinical practice guidelines in oncology. J Natl Compr Canc Netw (2019) 17(7):855–83. doi: 10.6004/jnccn.2019.0033 31319389

[20] Minamide J Aoyama N Koizumi H Yoneyama K Hoshino S Kamiya J. Postoperative complications in patients of esophageal cancer after neoadjuvant chemotherapy. Jpn J Thorac Cardiovasc Surg Off Publ Jpn Assoc Thorac Surg = Nihon KyobuGeka Gakkai Zasshi (1999) 47(11):542–5. doi: 10.1007/bf03218059 10614093

[21] Li H Yang S Zhang Y Xiang J Chen H. Thoracic recurrent laryngeal lymph node metastases predict cervical node metastases and benefit from three-field dissection in selected patients with thoracic esophageal squamous cell carcinoma. J Surg Oncol (2012) 105(6):548–52. doi: 10.1002/jso.22148 22105736

[22] Rice TW Patil DT Blackstone EH. 8th edition ajcc/uicc staging of cancers of the esophagus and esophagogastric junction: application to clinical practice. Ann Cardiothorac Surg (2017) 6(2):119–30. doi: 10.21037/acs.2017.03.14 PMC5387145 28447000

[23] Dueck AC Mendoza TR Mitchell SA Reeve BB Castro KM Rogak LJ. Validity and reliability of the us national cancer institute's patient-reported outcomes version of the common terminology criteria for adverse events (pro-ctcae). JAMA Oncol (2015) 1(8):1051–9. doi: 10.1001/jamaoncol.2015.2639 PMC4857599 26270597

[24] Zhang W Yan C Zhang T Chen X Dong J Zhao J. Addition of camrelizumab to docetaxel, cisplatin, and radiation therapy in patients with locally advanced esophageal squamous cell carcinoma: a phase 1b study. Oncoimmunology (2021) 10(1):1971418. doi: 10.1080/2162402X.2021.1971418 34616588 PMC8489938

[25] Zhou YX Chen P Sun YT Zhang B Qiu MZ. Comparison of pd-1 inhibitors in patients with advanced esophageal squamous cell carcinoma in the second-line setting. Front Oncol (2021) 11:698732. doi: 10.3389/fonc.2021.698732 34621668 PMC8490758

[26] Liu J Li J Lin W Shao D Depypere L Zhang Z. Neoadjuvant camrelizumab plus chemotherapy for resectable, locally advanced esophageal squamous cell carcinoma (nic-escc2019): a multicenter, phase 2 study. Int J Cancer (2022) 151(1):128–37. doi: 10.1002/ijc.33976 35188268

[27] Liu J Yang Y Liu Z Fu X Cai X Li H. Multicenter, single-arm, phase ii trial of camrelizumab and chemotherapy as neoadjuvant treatment for locally advanced esophageal squamous cell carcinoma. J Immunother Cancer (2022) 10(3):e004291. doi: 10.1136/jitc-2021-004291 PMC8961177 35338088

[28] Yang G Su X Yang H Luo G Gao C Zheng Y. Neoadjuvant programmed death-1 blockade plus chemotherapy in locally advanced esophageal squamous cell carcinoma. Ann Transl Med (2021) 9(15):1254. doi: 10.21037/atm-21-3352 34532391 PMC8421958

[29] Yang W Xing X Yeung SJ Wang S Chen W Bao Y. Neoadjuvant programmed cell death 1 blockade combined with chemotherapy for resectable esophageal squamous cell carcinoma. J Immunother Cancer (2022) 10(1):e003497. doi: 10.1136/jitc-2021-003497 PMC8756283 35022193

[30] Yang Y Zhu L Cheng Y Liu Z Cai X Shao J. Three-arm phase ii trial comparing camrelizumab plus chemotherapy versus camrelizumab plus chemoradiation versus chemoradiation as preoperative treatment for locally advanced esophageal squamous cell carcinoma (nice-2 study). BMC Cancer (2022) 22(1):506. doi: 10.1186/s12885-022-09573-6 35524205 PMC9074348

[31] Yang P Zhou X Yang X Wang Y Sun T Feng S. Neoadjuvant camrelizumab plus chemotherapy in treating locally advanced esophageal squamous cell carcinoma patients: a pilot study. World J Surg Oncol (2021) 19(1):333. doi: 10.1186/s12957-021-02446-5 34809658 PMC8609728

[32] Wu Z Zheng Q Chen H Xiang J Hu H Li H. Efficacy and safety of neoadjuvant chemotherapy and immunotherapy in locally resectable advanced esophageal squamous cell carcinoma. J Thorac Dis (2021) 13(6):3518–28. doi: 10.21037/jtd-21-340 PMC8264718 34277047

[33] Shang X Zhao G Liang F Zhang C Zhang W Liu L. Safety and effectiveness of pembrolizumab combined with paclitaxel and cisplatin as neoadjuvant therapy followed by surgery for locally advanced resectable (stage iii) esophageal squamous cell carcinoma: a study protocol for a prospective, single-arm, single-center, open-label, phase-ii trial (keystone-001). Ann Transl Med (2022) 10(4):229. doi: 10.21037/atm-22-513 35280363 PMC8908169

[34] Shang X Zhang W Zhao G Liang F Zhang C Yue J. Pembrolizumab combined with neoadjuvant chemotherapy versus neoadjuvant chemoradiotherapy followed by surgery for locally advanced oesophageal squamous cell carcinoma: protocol for a multicentre, prospective, randomized-controlled, phase iii clinical study (keystone-002). Front Oncol (2022) 12:831345. doi: 10.3389/fonc.2022.831345 35433421 PMC9008846

[35] Huang B Shi H Gong X Yu J Xiao C Zhou B. Comparison of efficacy and safety between pembrolizumab combined with chemotherapy and simple chemotherapy in neoadjuvant therapy for esophageal squamous cell carcinoma. J Gastrointest Oncol (2021) 12(5):2013–21. doi: 10.21037/jgo-21-610 PMC8576253 34790369

[36] Gao L Lu J Zhang P Hong ZN Kang M. Toripalimab combined with docetaxel and cisplatin neoadjuvant therapy for locally advanced esophageal squamous cell carcinoma: a single-center, single-arm clinical trial (esonict-2). J Gastrointest Oncol (2022) 13(2):478–87. doi: 10.21037/jgo-22-131 PMC9086050 35557591

[37] Xing W Zhao L Fu X Liang G Zhang Y Yuan D. A phase ii, single-centre trial of neoadjuvant toripalimab plus chemotherapy in locally advanced esophageal squamous cell carcinoma. J Thorac Dis (2020) 12(11):6861–7. doi: 10.21037/jtd-20-2198 PMC7711391 33282388

[38] Zheng Y Liu XB Sun HB Xu J Shen S Ba YF. A phase iii study on neoadjuvant chemotherapy versus neoadjuvant toripalimab plus chemotherapy for locally advanced esophageal squamous cell carcinoma: henan cancer hospital thoracic oncology group 1909 (hchtog1909). Ann Transl Med (2021) 9(1):73. doi: 10.21037/atm-20-5404 33553366 PMC7859818

[39] He W Leng X Mao T Luo X Zhou L Yan J. Toripalimab plus paclitaxel and carboplatin as neoadjuvant therapy in locally advanced resectable esophageal squamous cell carcinoma. Oncologist (2022) 27(1):e18–28. doi: 10.1093/oncolo/oyab011 PMC8842349 35305102

[40] Zhang Z Hong ZN Xie S Lin W Lin Y Zhu J. Neoadjuvant sintilimab plus chemotherapy for locally advanced esophageal squamous cell carcinoma: a single-arm, single-center, phase 2 trial (esonict-1). Ann Transl Med (2021) 9(21):1623. doi: 10.21037/atm-21-5381 34926667 PMC8640906

[41] Klevebro F Tsekrekos A Low D Lundell L Vieth M Detlefsen S. Relevant issues in tumor regression grading of histopathological response to neoadjuvant treatment in adenocarcinomas of the esophagus and gastroesophageal junction. Dis Esophagus (2020) 33(6):doaa005. doi: 10.1093/dote/doaa005 PMC7273185 32141500

[42] Langer R Becker K. Tumor regression grading of gastrointestinal cancers after neoadjuvant therapy. Virchows Arch (2018) 472(2):175–86. doi: 10.1007/s00428-017-2232-x 28918544

[43] Jia R Xiao W Zhang H Yu Z. Comparative study of treatment options and construction nomograms to predict survival for early-stage esophageal cancer: a population-based study. Scand J Gastroenterol (2021) 56(6):635–46. doi: 10.1080/00365521.2021.1910997 33872097

[44] Qi Z Hu Y Qiu R Li J Li Y He M. Survival risk prediction model for patients with pt1-3 n0m0 esophageal squamous cell carcinoma after R0 esophagectomy with two-field lymphadenectomy for therapeutic purposes. J Cardiothorac Surg (2021) 16(1):121. doi: 10.1186/s13019-021-01503-0 33933129 PMC8088719

[45] Xie SH Santoni G Malberg K Lagergren P Lagergren J. Prediction model of long-term survival after esophageal cancer surgery. Ann Surg (2021) 273(5):933–9. doi: 10.1097/SLA.0000000000003431 33824250

[46] Zhang G Guo X Yuan L Gao Z Li J Li X. Examined lymph node count is not associated with prognosis in elderly patients with pn0 thoracic esophageal cancer. Med (Baltimore) (2021) 100(2):e24100. doi: 10.1097/MD.0000000000024100 PMC7808502 33466178

[47] Hong ZN Weng K Peng K Chen Z Lin J Kang M. Neoadjuvant immunotherapy combined chemotherapy followed by surgery versus surgery alone for locally advanced esophageal squamous cell carcinoma: a propensity score-matched study. Front Oncol (2021) 11:797426. doi: 10.3389/fonc.2021.797426 34970498 PMC8712481

[48] Naidoo J Wang X Woo KM Iyriboz T Halpenny D Cunningham J. Pneumonitis in patients treated with anti-programmed death-1/programmed death ligand 1 therapy. J Clin Oncol (2017) 35(7):709–17. doi: 10.1200/JCO.2016.68.2005 PMC5559901 27646942

[49] Wu LL Zhong JD Zhu JL Kang L Huang YY Lin P. Postoperative survival effect of the number of examined lymph nodes on esophageal squamous cell carcinoma with pathological stage T1-3n0m0. BMC Cancer (2022) 22(1):118. doi: 10.1186/s12885-022-09207-x 35090428 PMC8800278

[50] Shang QX Yang YS Xu LY Yang H Li Y Li Y. Prognostic role of nodal skip metastasis in thoracic esophageal squamous cell carcinoma: a large-scale multicenter study. Ann Surg Oncol (2021) 28(11):6341–52. doi: 10.1245/s10434-020-09509-z 33738720

[51] Liu G Han Y Peng L Wang K Fan Y. Reliability and safety of minimally invasive esophagectomy after neoadjuvant chemoradiation: a retrospective study. J Cardiothorac Surg (2019) 14(1):97. doi: 10.1186/s13019-019-0920-0 31138245 PMC6537410

[52] Sihag S Ku GY Tan KS Nussenzweig S Wu A Janjigian YY. Safety and feasibility of esophagectomy following combined immunotherapy and chemoradiotherapy for esophageal cancer. J Thorac Cardiovasc Surg (2021) 161(3):836–43.e1. doi: 10.1016/j.jtcvs.2020.11.106 PMC7889638 33485662

